# The impact of disrespectful care during childbirth on maternal mental health: postpartum depression and suicidal ideation 6–9 months after birth

**DOI:** 10.3389/fgwh.2026.1702931

**Published:** 2026-04-23

**Authors:** Ana Ballesta-Castillejos, Sandra Martínez-Rodríguez, Inmaculada Ortiz-Esquinas, Juan Miguel Martínez-Galiano, Victoria Mazoteras-Pardo, Antonio Hernández-Martínez

**Affiliations:** 1 Department of Nursing, Faculty of Nursing of Albacete, University of Castilla-La Mancha, Ciudad Real, Spain; 2 Instituto de Investigación Sanitaria de Castilla la Mancha (IDISCAM), Hospital Nacional de Parapléjicos, Toledo, Spain; 3 Department of Nursing, Faculty of Nursing of Ciudad Real, University of Castilla-La Mancha, Ciudad Real, Spain; 4 Hospital Universitario Reina Sofia de Córdoba, Cordoba, Spain; 5 Department of Nursing of University of Jaen, Jaén, Spain; 6 CIBER de Epidemiología y Salud Pública (CIBERESP), Madrid, Spain

**Keywords:** disrespect, mistreatment, obstetric violence, postpartum depression, suicide

## Abstract

**Introduction:**

Postpartum depression is a common mental health disorder that has negative consequences for the mother, the family environment, and the newborn's development. In severe cases, it can be accompanied by suicidal ideation, posing a significant risk to maternal health and the mother-child bond. Recently, studies have begun to investigate how obstetric violence during childbirth, which many women experience, can negatively impact their mental health. The objective of this study was to assess the relationship between inappropriate or disrespectful treatment during birth and the presence of postpartum depression and suicidal ideation six to nine months after delivery.

**Methods:**

A cross-sectional observational study was conducted with women who were 6–9 months postpartum in Spain. The Edinburgh Postnatal Depression Scale was used to determine the risk of postpartum depression, and item 10 was used to identify suicide risk. The Childbirth Abuse and Respect Assessment-Maternal Questionnaire was used to assess women's experiences of inappropriate treatment during childbirth. To evaluate social support, the family APGAR, the Medical Outcomes Study Social Support Survey, and the Woman Abuse Screening Tool were used. Adjusted ORs and their 95% CI were calculated. 341 women participated.

**Results:**

Two risk factors associated with postpartum depression were identified: hospital readmission (OR: 7.19; 95% CI: 1.56–33.15) and elevated scores on the Childbirth Abuse and Respect Evaluation-Maternal Questionnaire (CARE-MQ). Regarding the latter, a dose-response pattern was observed: scores at the 70th–90th percentile were associated with a moderate increase in risk (OR: 2.48; 95% CI: 1.05–5.88), while scores above the 90th percentile were associated with a substantially greater risk (OR: 5.26; 95% CI: 2.35–11.79). Regarding suicidal ideation, hospitalization of the newborn (OR: 2.66; 95% CI: 1.14–6.18) was identified as a risk factor, while high scores on the family APGAR test (OR: 0.86; 95% CI: 0.80–0.92) acted as a protective factor for its occurrence.

**Conclusions:**

Inadequate care during birth has been identified as a significant risk factor for the development of postpartum depression between 6 and 9 months after birth. However, this association has not been observed in relation to suicidal ideation.

## 1 Introduction

Depressive symptoms are common after childbirth (1). In Western countries, the prevalence of postpartum depression (PPD) ranges between 10% and 15% during the first year postpartum (2). The recent meta-analysis by Liu et al. observed a prevalence of PPD in the first year between 5% and 26%, with an overall prevalence of 14% (3), a figure similar to that reported by the meta-analysis by Shorey et al., which sets this prevalence at 17% (4).

Among the emotional changes that may occur in the postpartum period, it is important to differentiate between the transient symptoms that usually appear in the first 10–12 days postpartum and postpartum depression (PPD). Transient symptoms tend to resolve spontaneously, do not significantly interfere with daily functioning, and do not require medical treatment, whereas PPD is a mental disorder considered a major depressive disorder that does require intervention and treatment (5).

PPD affects not only the mother but also the family environment. It impacts the mother's physical and mental well-being, impairs the mother-child relationship, generates family conflicts, and increases the risk of depressive episodes in the following 5 years (6, 7). Regarding the newborn, a higher likelihood of developing behavioral problems and even worse long-term academic outcomes has been observed (8).

In some cases, postpartum depression has been associated with other disorders such as suicidal ideation (9, 10), which represents a serious risk factor for suicide attempts or even completed suicide (11). The estimated prevalence of suicidal ideation in the postnatal period is 7%, with the highest prevalence occurring in the first 4 months after birth (12). In fact, there is growing evidence that the prevalence of suicidal ideation in the perinatal period is higher than in the general population (12). The impact of these data on a clinical level is significant, as it has been estimated that postpartum depression is related to approximately 20% of maternal deaths after childbirth, making it a serious public health problem (13). Furthermore, suicidal ideation has been linked to issues in mother-child bonding (14) and in children's cognitive development, such as speech and motor skills problems, and even an increased risk of suicide in adulthood (14, 15).

On the other hand, recent attention has begun to be paid to the quality of care women receive during pregnancy and childbirth, and how this may influence their subsequent mental health (16–18).

Recent studies have observed that approximately 60% of women report having experienced obstetric violence during childbirth globally, with the figure rising to 67.4% in Spain (19). Obstetric violence is understood as a form of gender-based violence perpetrated against women during pregnancy, childbirth, and the postpartum period, which violates their human and reproductive rights (20).

Obstetric abuse and mistreatment during childbirth treatment directly impacts their mental health, promoting the development of conditions such as post-traumatic stress disorder or postpartum depression (21–23). However, studies linking inappropriate treatment during birth with postpartum depression or suicidal ideation are scarce, and those that do exist have significant limitations, such as the lack of validated tools to assess this treatment. Furthermore, it is unknown whether this experience of inappropriate treatment during birth can influence the risk of developing PPD and suicidal ideation in the medium and long term.

For all these reasons, in this study, we aimed to assess the relationship between inappropriate or disrespectful treatment during birth and the presence of postpartum depression and suicidal ideation six to nine months after birth.

## 2 Materials and methods

### 2.1 Study design, setting and participant selection

This study employed a cross-sectional observational design with a sample of postpartum women in Spain who completed the questionnaire between 6 and 9 months postpartum.

Data were collected nationwide through a community-based, population-level online survey. The study was distributed through pregnancy, childbirth, postpartum, and breastfeeding support associations and groups across Spain, using mailing lists and social media platforms.

### 2.2 Research participants selection

A non-probabilistic convenience sampling strategy was used. Women were recruited through collaboration with maternal and breastfeeding associations, which disseminated the study invitation via mailing lists and social media platforms. The invitation included a brief explanation of the study objectives and a link to the online questionnaire. Participation was entirely voluntary and anonymous.

Inclusion criteria were women aged 18 years or older, able to read and write in Spanish, no prior diagnosis of any mental health condition before pregnancy, and having given birth within the previous 6–9 months at the time of completing the questionnaire.

The exclusion criteria were women under 18 years of age, who did not speak or did not know the Spanish language (language barrier), and those who had a diagnosis of any psychiatric disorders prior to pregnancy.

Eligibility was assessed through screening questions placed at the beginning of the online questionnaire. Women who did not meet the inclusion criteria were automatically redirected and excluded from participation before accessing the full instrument.

The maximum modeling criterion was used to estimate the sample size, which requires including 10 research participants for each independent variable (24). Considering that the prevalence of PPD risk can reach up to 26% (25), a minimum of 100 women at risk of PPD were required to include 9 independent variables in the multivariate model, and globally, about 346 women.

### 2.3 Data collection procedure

An online self-administered questionnaire was developed. The questionnaire was distributed through official channels of pregnancy, birth, postpartum, and breastfeeding support associations across Spain. Associations were contacted directly by the research team and agreed to share the study invitation with their members via internal mailing lists and social media groups. Participants accessed the questionnaire through a direct hyperlink included in the invitation. Before accessing the questionnaire, participants were presented with the study information sheet, including objectives, voluntary nature of participation, confidentiality, and data protection measures. Electronic informed consent was obtained prior to accessing any study items. Eligibility screening questions appeared first; those not meeting inclusion criteria were redirected and excluded.

### 2.4 Information sources

An online questionnaire was developed and distributed to associations related to pregnancy, birth, and postpartum, as well as to breastfeeding support groups throughout Spain. The questionnaire included sociodemographic variables, obstetric history, variables related to the most recent birth, obstetric practices performed, and neonatal outcomes.

Various tools were included in this questionnaire:

- Childbirth Abuse and Respect Evaluation- Maternal Questionnaire” (CARE-MQ) (26).

This tool is made up of Likert-type questions about different practices and/or situations that can be related to abuse and lack of respect during childbirth. The instrument consists of four components (“Emotional Abuse”, “Inadequate Professionalism”, “Physical Abuse”, and “Lost contact”). Each of these components contains a series of items related to it, for example: requesting consent to perform examinations or procedures, providing information, ensuring the woman's privacy, or carrying out clinical practices based on evidence, among others. The possible answers are: “It did not occur during my birth” (0 points), “It occurred, but it did not affect me” (1 point), “It occurred and it affected me a little” (2 points), and “It occurred and it affected me a lot” (3 points). The total score ranged from 0 points to 60 points. The scores can be categorized based on the distribution of percentiles (≤50th percentile, 51–75th percentile, 75–90th percentile, >90th percentile). The tool has shown adequate internal consistency (Cronbach's α value was 0.903) and excellent temporal stability in test-retest [the coefficient of intraclass correlation of absolute agreement after administering the questionnaire one week later was 0.927 (95% CI: 0.85–0.97)].

- Edinburgh Postnatal Depression Scale (EPDS).

This scale is made up of 10 items that are self-completed and are used to detect postpartum depression specifically. This has been validated in the Spanish population during the postpartum period (27). A score of ≥12 points was considered PPD risk following the recommendations of Gibson et al. (28), observing predictive value for positive 61% and for negatives 96% and an LR+ of 10.3 and LR− of 0.35. A score of 1, 2, or 3 on item 10 (“The thought of harming myself has occurred to me”) indicates a risk of suicidality.

- Family Apgar

The family Apgar assesses an individual's perceived satisfaction with five dimensions of family dynamics: adaptation, participation, growth, affection, and resolution. The scale comprises five items with three possible Likert-type responses: “Almost never” (0 points), “sometimes” (1 point), and “almost always” (2 points). The total score ranges from 0 to 10 points:

- 0–3 points. Indicates low satisfaction or a dysfunctional family.
- 4–6 points. Indicates medium satisfaction or possible dysfunction.
- 7–10 points. Indicates high satisfaction in a functional family.

This scale has been validated in the Spanish population, demonstrating good internal consistency (Cronbach's α ranging from 0.84 to 0.86) and adequate construct validity.

- Medical Outcomes Study Social Support Survey (MOS-SSS) (29).

The MOS-SSS scale is a self-administered instrument designed to assess perceived social support. It consists of 19 items that explore various dimensions of social support, including emotional, instrumental, and affective support, as well as positive social interaction. The full version of the scale has been validated in the Spanish population through a descriptive study with a sample of 903 patients. The results showed excellent internal reliability (Cronbach's *α* = 0.96), as well as a consistent unidimensional structure confirmed by exploratory and confirmatory factor analysis (KMO = 0.904; CFI = 0.95; TLI = 0.97; SRMR = 0.05) (30).

- Woman Abuse Screening Tool (WAST)

The short version of the Woman Abuse Screening Tool (WAST) (31) is a self-administered, two-item scale developed to detect gender-based violence in women in primary care settings. This version has been validated in the Spanish population. The instrument explores relationship tension and difficulty resolving conflicts. Responses are scored on a three-point Likert-type scale:

- 0 points: no tension, no difficulty.
- 1 point: some tension, some difficulty.
- 2 points: significant tension, significant difficulty.

A score of 2 is considered a risk of abuse.

### 2.5 Statistical analysis

For sociodemographic and clinical data, absolute and relative frequencies were used to describe qualitative variables, and the mean and standard deviation (SD) were used to describe quantitative variables.

Next, the relationship between the experience of abuse and disrespect during childbirth (CARE-MQ) and the risk of PPD (score ≥12 points) and positive scores on question number 10 of the EPDS was analyzed in a bivariate and multivariate manner.

This scale has been categorized by percentiles because the distribution of the scores does not follow a normal distribution, and it is advisable to use statistics based on ordinal scales, such as percentiles. In addition, these cut-off points allow us to have a more practice-oriented view and determine from which score an increase in the risk of the events studied occurs.

Sociodemographic and clinical variables identified in the literature as potentially influencing the risk of PPD were introduced as control variables. Crude (OR) and adjusted Odds Ratios (aOR) were estimated with their respective 95% confidence intervals (95%CI).

Additionally, three validated psychosocial scales were included in the analysis: the family Apgar scale, which measures perceived family support; the MOS Social Support Survey, which assesses perceived social support; and the Worries About Screening questionnaire (WAST), which evaluates concern or anxiety related to perinatal screening. These three instruments were treated as continuous quantitative variables. Descriptive statistics were calculated using means and standard deviations.

In the multivariate analysis, all variables from the bivariate analysis were included, regardless of their significance level. Variables that remained in the multivariate model were selected using the backward stepwise elimination procedure (RV in SPSS). The automatic selection model was used with PIN values (0.05), POUT values (0.10), LCON (0), and BCON (0.001). PIN specifies the minimum probability that a variable can have of entering the analysis, and POUT specifies the maximum probability that a variable can have and not be eliminated from the model. BCON specifies the change in parameter estimates to end the iteration, and LCON specifies the percentage change in the log likelihood ratio to end the iteration.

### 2.6 Ethical considerations

Approval was obtained from the clinical research ethics committees of the following hospitals: Mancha-Centro Hospital (197-C), Reina Sofia University Hospital of Córdoba (5615), and University Hospital of Ciudad Real (C−600). All participants received written information about the study objectives, confidentiality, voluntary participation, and data protection. Electronic informed consent was obtained prior to accessing the questionnaire.

Participants were required to provide a contact telephone number or email address only for potential follow-up contact if clarification was needed; these data were handled in accordance with data protection regulations.

## 3 Results

### 3.1 Sample characteristics

A sample of 341 women was obtained. The mean age was 33.8 years (SD: 4.23). 91.5% (*n* = 312) had a planned pregnancy, and 80.1% (*n* = 273) were primiparous. 56.3% (*n* = 192) experienced a spontaneous onset of labour, and 57.2% (*n* = 195) had a spontaneous vaginal birth. Furthermore, 62.8% (*n* = 214) had skin-to-skin contact with their newborn for more than 120 min, and 73.6% (*n* = 251) were discharged from the hospital exclusively breastfeeding.

Regarding the CARE-MQ percentiles, almost half of the participants [49.6% (*n* = 169)] were in the ≤50th percentile (≤3 points) (Table 1).

**Table 1:** Characteristics of the sample.

Variable	*N* (%) *N* = 341	Variable	*N* (%) *N* = 341
Maternal age mean (SD)	33.83 (4.22)	General anesthesia	
Income level		No	325 (95.3)
<2,000 euros	57 (16.7)	Yes	16 (4.7)
2,000–2,999 euros	110 (32.3)	Type of birth	
≥3,000 euros	174 (51.0)	Normal birth	195 (57.2)
Planned pregnancy		Instrumental	65 (19.1)
No	29 (8.5)	Elective CS	11 (3.2)
Yes	312 (91.5)	Emergency CS	70 (20.5)
Antenatal education		Episiotomy	
No	49 (14.4)	No	273 (80.1)
Yes	292 (85.6)	Yes	68 (19.9)
Birth plan		Severe tear	
No	105 (30.8)	No	325 (95.3)
Yes, not respected	65 (19.1)	Yes	16 (4.7)
Yes, and respected	171 (50.1)	Maternal ICU admission	
Parity		No	337 (98.8)
Primiparous	273 (80.1)	Yes	4 (1.2)
Multiparous	68 (19.9)	Skin-to-skin	
Induction of labor		Less than 120 min	127 (37.2)
No	192 (56.3)	Yes, and at least 120 min	214 (62.8)
Yes	149 (43.7)	Neonatal admission	
Gestational diabetes		No	288 (84.5)
No	311 (91.2)	Yes	53 (15.5)
Yes	30 (8.8)	Live birth	
APP		No	3 (0.9)
No	323 (94.7)	Yes	338 (99.1)
Yes	18 (5.3)	Regional analgesia	
Problems during childbirth		No	90 (26.4)
No	251 (73.6)	Yes	251 (73.6)
Yes	90 (26.4)	Postnatal surgical intervention	
Mother Hospital readmission		No	316 (92.7)
No	331 (97.1)	Yes	25 (7.3)
Yes	10 (2.9)	Risk of PPD	
CARE-MQ (percentiles)		No	275 (80.6)
*P* ≤50 (≤3 points)	169 (49.6)	Yes	66 (19.4)
*P* 51–75 (3–9 points)	72 (21.1)	Risk of suicidal ideation	
*P* 76–90 (10–21 points)	50 (14.7)	No	308 (90.3)
*P* > 90 (≥22 points)	50 (14.7)	Yes	33 (9.7)
Family Apgar (SD)	15.15 (0.24)	MOS-SSS (SD)	82.15 (0.78)
Exclusive breastfeeding at hospital discharge		WAST (SD)	1.03 (0.05)
No	90 (26.4)		
Yes	251 (73.6)		

### 3.2 Risk of postpartum depression

The relationship between the risk of PPD and various sociodemographic, clinical, and birth-related variables was then analyzed using bivariate analysis. As shown in Table 2, several obstetric and clinical factors were associated with a higher likelihood of developing PPD (*p* < 0.05). Regarding childbirth experience, higher scores on the CARE-MQ scale (grouped by percentiles) were associated with increased PPD risk. Additionally, a higher likelihood of PPD was observed when the birth plan was not followed, when labour was induced and when complications arose during birth. Concerning analgesia and mode of birth, the administration of both regional and general anesthesia, instrumental births, and emergency caesarean sections were also associated with a higher likelihood of PPD. Finally, regarding neonatal and maternal outcomes, neonatal admission, the need for maternal surgery, maternal readmission after discharge and high scores on the WAST scale were all associated with an increased risk of PPD.

**Table 2:** Factors associated with the risk of PPD—bivariate and multivariate analysis.

Variable	Risk of PPD		Bivariate analysis		Multivariate analysis	
	No *n* (%) *N* (275)	Yes *n* (%) *N* (66)	aOR 95% CI	*P* value	aOR 95% CI	*P* value
Maternal age Mean (SD)	33.7 (4.17)	34.0 (4.47)	1.01 (0.95–1.08)	0.594		
Income level				0.016		
<2,000 euros	42 (73.7)	15 (26.3)	1			
2,000–3,000 euros	82 (74.5)	28 (25.5)	0.95 (0.46–1.98)	0.904		
>3,000 euros	151 (86.8)	23 (13.2)	**0.42** (**0.20–0.88)**	**0**.**023**		
Birth plan				**<0**.**001**		
No	87 (82.9)	18 (17.1)	1			
Yes, not respected	39 (60.0)	26 (40.0)	**3.22** (**1.58–6.55)**	**<0**.**001**		
Yes, and respected	149 (87.1)	22 (12.9)	0.71 (0.36–1.40)	0.329		
Gestational age				0.275		
Term	260 (81.3)	60 (18.8)	1			
Preterm	15 (71.4)	6 (28.6)	1.73 (0.64–4.65)			
Parity				0.459		
Primiparous	218 (79.9)	55 (20.1)	1			
Multiparous	57 (83.8)	11 (16.2)	0.76 (0.37–1.55)			
Induction of labor				**0**.**015**		
No	166 (86.5)	26 (13.5)	1			
Yes	109 (73.2)	40 (26.8)	**1.37** (**1.06–1.76)**			
Problems during childbirth				**<0**.**001**		
No	215 (85.7)	36 (14.3)	1			
Yes	60 (66.7)	30 (33.3)	**2.98** (**1.70–5.24)**			
Live newborn				0.547		
No	2 (66.7)	1 (33.3)	1			
Yes	273 (80.8)	65 (19.2)	0.47 (0.04–5.33)			
Type of birth				**<0**.**001**		
Vaginal birth	172 (88.2)	23 (11.8)	1			
Instrumental vaginal birth	49 (75.4)	16 (24.6)	**1.96** (**1.44–2.67)**	**<0**.**001**		
Elective CS	5 (45.5)	6 (54.4)	0.78 (0.36–1.67)	0.524		
Emergency CS	49 (70.0)	21 (30.0)	**1.78** (**1.28–2.46)**	**<0**.**001**		
Severe tear				0.225		
No	264 (81.2)	61 (18.8)	1			
Yes	11 (68.8)	5 (31.3)	1.96 (0.65–5.87)			
ICU admission				0.775		
No	272 (80.7)	65 (19.3)	1			
Yes	3 (75.0)	1 (25.0)	1.39 (0.14–13.62)			
Skin-to-skin				**<0**.**001**		
Less than 120 min	88 (69.3)	39 (30.7)	**1**			
Yes, and at least 120 min	187 (87.4)	27 (12.6)	**0.32** (**0.18–0.56)**			
Neonatal Admission				**0**.**012**		0.086
No	239 (83.0)	49 (17.0)	**1**		1	
Yes	36 (67.9)	17 (32.1)	**2.30** (**1.19–4.42)**		1.91 (0.91–4.02)	
Exclusive breastfeeding at hospital discharge				**<0**.**001**		**0**.**039**
No	61 (67.7)	29 (32.2)	**1**		**1**	
Yes	214 (85.3)	37 (14.7)	**0.36** (**0.20–0.63)**		**0.50** (**0.26–0.96)**	
Postnatal surgical intervention				**0**.**009**		
No	260 (82.3)	56 (17.7)	**1**			
Yes	15 (60.0)	10 (40.0)	**3.09** (**1.32–3.09)**			
Hospital readmission				**0**.**004**		**0**.**011**
No	271 (81.9)	60 (18.1)	**1**		**1**	
Yes	4 (40.0)	6 (60.0)	**6.77** (**1.85–24.75)**		**7.19** (**1.56–33.13)**	
CARE-MQ (percentiles)				**<0**.**001**		**<0**.**001**
*P* ≤50 (≤3 points)	153 (90.5)	16 (9.5)	1		1	
*P* 51–75 (3–9 points)	57 (79.2)	15 (20.8)	2.51 (1.16–5.42)	0.018	1.65 (0.72–3.80)	0.234
*P* 76–90 (10–21 points)	36 (72.0)	14 (28.0)	**3.72** (**1.66–8.03)**	**<0**.**001**	**2.48** (**1.05–5.88)**	**0**.**039**
*P* > 90 (≥22 points)	29 (58.0)	21 (42.0)	**6.92** (**3.23–14.83)**	**<0**.**001**	**5.27** (**2.35–11.80)**	**<0**.**001**
Family apgar mean (SD)	15.7 (4.19)	12.9 (5.36)	**0.88** (**0.83–0.93)**	**<0**.**001**		
MOS-SSS mean (SD)	83.9 (13.64)	74.8 (15.53)	**0.96** (**0.94–0.97)**	**<0**.**001**	**0.96** (**0.94–0.98)**	**<0**.**001**
WAST mean (SD)	0.96 (0.97)	1.36 (1.13)	**1.45** (**1.12–1.88)**	**0**.**004**		

PPD, postpartum depression ;SD, standard deviation; CI, confidence interval; OR, odds ratio; aOR, adjusted odds ratio; CS, cesarean.

Values in bold are statistically significant.

In contrast, some factors acted as protective factors, such as an income above 3,000 euros per month, skin-to-skin contact for more than 120 min after birth, or leaving the hospital with EBF, as well as higher family APGAR and MOS-SSS scores.

In the multivariate analysis of all the above factors, four factors were identified that were statistically significantly associated with the risk of postpartum depression between 6 and 9 months after birth, after controlling for confounding factors. First, hospital readmission after birth increased the risk of developing postpartum depression (aOR: 7.19; 95% CI: 1.56–33.15). On the other hand, high scores on the CARE-MQ scale were also associated with an increased risk; specifically, being in the 76th−90th percentile was associated with a 2.48-fold (95% CI: 1.05–5.88) increase in the likelihood, while reaching a percentile above the 90th increased the risk by 5.26-fold (95% CI: 2.35–11.79) compared to women below the 50th percentile.

In contrast, exclusive breastfeeding at discharge was shown to have a protective effect, reducing the likelihood of postpartum depression by half (aOR: 0.50; 95% CI: 0.26–0.96). Similarly, higher scores on the MOS-SSS social support scale were associated with a significant risk reduction (aOR: 0.96; 95% CI: 0.94–0.98), indicating a protective effect that reduces the likelihood of depression by 4% for each additional point.

### 3.3 Risk of suicidal ideation

An analysis was then conducted between the risk of suicidal ideation and various sociodemographic and clinical factors 6–9 months postpartum (Table 3). According to the bivariate analysis, a significant association was observed between higher scores on the CARE-MQ scale and a higher risk of suicidal ideation. According to the bivariate analysis, a significant association was observed between higher scores on the CARE-MQ scale and a higher risk of suicidal ideation. Specifically, women scoring in the 76th–90th percentile (10–21 points) showed a significantly higher risk of suicidal ideation compared to those in the ≤50th percentile (aOR: 3.38; 95% CI: 1.23–9.30), as did women scoring above the 90th percentile (≥22 points) (aOR: 2.89; 95% CI: 1.01–8.21). Other factors associated with a higher risk of suicidal ideation included having experienced complications during birth, the newborn requiring neonatal admission, and high scores on the WAST scale.

**Table 3:** Factors associated with the risk of suicidal ideation (positive on item 10 EPDS)—bivariate and multivariate analysis.

Variable	Risk of suicidal ideation		Bivariate analysis		Multivariate analysis	
	No *n* (%) *N* (308)	Yes *n* (%) *N* (33)	aOR 95% CI	*P* value	aOR 95% CI	*P* value
Maternal age Mean (SD)	33.8 (4.14)	33.8 (4.96)	0.58 (−1.44 to 0.83)	0.595		
Income level				0.071		
<2,000 euros	47 (82.5)	10 (17.5)	1			
2,000–3,000 euros	99 (90.0)	11 (10.0)	0.52 (0.20–1.31)	0.168		
>3,000 euros	162 (93.2)	12 (6.9)	**0.34** (**0.14–0.85)**	**0**.**022**		
Birth plan				**0**.**026**		
No	95 (90.5)	10 (9.5)	1			
Yes, not respected	53 (81.5)	12 (18.5)	2.15 (0.87–5.31)	0.097		
Yes, and respected	160 (93.6)	11 (6.4)	0.65 (0.26–1.59)	0.350		
Gestational age				0.465		
Term	260 (81.3)	60 (18.8)	1			
Preterm	15 (71.4)	6 (26.8)	1.61 (0.44–5.78)			
Parity				0.516		
Primiparous	248 (90.8)	25 (9.2)	1			
Multiparous	60 (88.2)	8 (11.8)	1.32 (0.56–3.07)			
Induction of labor				0.560		
No	175 (91.1)	17 (8.9)	1			
Yes	133 (89.3)	16 (10.7)	1.23 (0.60–2.54)			
Problems during childbirth				**0**.**011**		
No	233 (92.8)	18 (7.2)	**1**			
Yes	75 (83.3)	15 (16.7)	**2.58** (**1.24–5.38)**			
Live newborn						
No	3 (100)	0 (0.0)	1			
Yes	305 (90.2)	33 (9.8)	Not calculated			
Type of birth				0.207		
Normal birth	177 (90.8)	18 (9.2)	1			
Instrumental	61 (93.8)	4 (6.2)	0.64 (0.21–1.98)	0.443		
Elective CS	8 (72.7)	3 (27.3)	3.68 (0.89–15.14)	0.070		
Emergency CS	62 (88.6)	8 (11.4)	1.26 (0.52–3.06)	0.597		
Severe tear				0.697		
No	294 (90.5)	31 (9.5)	1			
Yes	14 (87.5)	2 (12.5)	1.35 (0.29–6.23)			
ICU admission				0.323		
No	305 (90.5)	32 (9.5)	1			
Yes	3 (75.0)	1 (25.0)	3.17 (0.32–31.44)			
Skin-to-skin				0.163		
Less than 120 min	111 (87.4)	16 (12.6)	1			
Yes, and at least 120 min	197 (92.1)	17 (7.9)	0.59 (0.29–1.23)			
Neonatal Admission				**0**.**017**		**0**.**023**
No	265 (92.0)	23 (8.0)	**1**		**1**	
Yes	43 (81.1)	10 (18.9)	**2.67** (**1.19–6.01)**		**2.66 (1.14–6.18)**	
Exclusive breastfeeding at hospital discharge				**0**.**011**		
No	75 (83.3)	15 (16.7)	**1**			
Yes	233 (92.8)	18 (7.2)	**0.38** (**0.18–0.80)**			
Postnatal surgical intervention				0.079		
No	288 (91.1)	28 (8.9)	1			
Yes	20 (80.0)	5 (20.0)	2.57 (0.89–7.37)			
Hospital readmission				0.972		
No	299 (90.3)	32 (9.7)	1			
Yes	9 (90.0)	1 (10.0)	1.03 (0.12–8.46)			
CARE-MQ (percentiles)				0.068		
*P* ≤50 (≤3 points)	160 (94.7)	9 (5.3)	1			
*P* 51–75 (3–9 points)	63 (87.5)	9 (12.5)	2.54 (0.96–0.69)	0.059		
*P* 76–90 (10–21 points)	42 (84.0)	8 (16.0)	**3.38** (**1.23–9.30)**	**0**.**018**		
*P* > 90 (≥22 points)	43 (86.0)	7 (14.0)	**2.89** (**1.01–8.21)**	**0**.**046**		
Family Apgar Mean (SD)	15 (4.32)	11.8 (5.50)	**0.86** (**0.80–0.92)**	**<0**.**001**	**0.86 (0.80–0.92)**	**<0**.**001**
MOS-SSS Mean (SD)	83.1 (14.01)	72.9 (15.47)	**0.96** (**0.94–0.98)**	**<0**.**001**		
WAST Mean (SD)	0.98 (0.05)	1.14 (0.20)	**1.59** (**1.14–2.21)**	**0**.**006**		

SD, standard deviation; CI, confidence interval; OR, odds ratio; aOR, adjusted odds ratio; NC, not calculated.

Values in bold are statistically significant.

Factors that decreased the likelihood of suicidal ideation were also identified: a monthly income above €3,000, a planned pregnancy, breastfeeding at discharge from the hospital, and higher scores on the family APGAR score and the MOS-SSS scale.

When performing multivariate analysis, only two factors were associated with the risk of suicidal ideation: neonatal admission, which increases the risk of suicidal ideation by 2.66 (aOR: 2.66; 95% CI: 1.14–6.18), and the family Apgar score (aOR: 0.86; 95%CI: 0.80– 0.92). In this sense, for each additional point in the Apgar test, the risk of suicidal ideation decreased by 14%.

## 4 Discussion

The percentage of women at risk for postpartum mental health disorders remains high, reaching figures close to 20%, even beyond six months after birth. This data highlights the persistence of the psychological impact of childbirth in the medium term. Among the factors identified as significantly associated with the risk of postpartum depression, high scores on the CARE-MQ scale stand out, indicating that the experience of abuse or disrespect during childbirth can have lasting emotional consequences beyond the immediate impact. This study identified three other factors associated with the risk of postpartum depression. On the one hand, the mother's hospital readmission is a risk factor. In contrast, we found two protective factors: exclusive breastfeeding upon hospital discharge and higher scores on the MOS-SSS social support scale, demonstrating the role of the environment in promoting care and emotional support in the prevention of this disorder.

Regarding the risk of suicidal ideation, neonatal admission was identified as the main risk factor, as it significantly increases the likelihood of suicidal thoughts developing in the mother. As a protective factor, higher scores on the family APGAR scale were associated with a lower risk, suggesting that a positive perception of family functioning may help regulate psychological distress in the postpartum period.

According to published meta-analyses, the average incidence of PPD in the first six months postpartum is approximately 12% (4); rising to between 14% (3) and 17% (4) over the first year. In the present study, the observed prevalence was 19.4%, a figure slightly higher than that reported in international meta-analyses conducted across diverse global contexts (4, 31). This difference may be partly attributable to the specific characteristics of the Spanish postpartum population, methodological heterogeneity across studies, and the use of different diagnostic instruments and cut-off scores.

These findings have important implications for the Spanish healthcare system. The fact that 19.4% of women in our sample were at risk for PPD six to nine months after birth — well beyond the immediate postpartum period — suggests that the psychological impact of childbirth is sustained and that current postnatal follow-up, which in Spain is typically concentrated in the first six weeks postpartum, may be insufficient to capture and address this burden.

The relationship between socioeconomic factors and depression is widely recognized, and evidence suggests that financial pressure is significantly associated with the onset of PPD (32). In our study, having a high socioeconomic status was identified as a protective factor against the development of this condition.

Existing studies have indicated that problems during birth, such as the need for instrumental birth or cesarean section, especially an emergency cesarean section, are associated with a higher likelihood of PPD. These findings are consistent with those obtained in the present study (33). Unplanned pregnancy has also been linked to an increased risk of PPD, which is consistent with our observation in this study, that having a planned pregnancy may reduce the risk of postpartum depression (34, 35).

Regarding breastfeeding, the literature is more extensive, and exclusive breastfeeding has been identified as a protective factor against postpartum depression, especially (36, 37). These results are consistent with those obtained in the present study; however, some discrepancies in this regard can also be found in the literature (38), possibly attributable to cultural differences.

Our study, in line with existing literature (3), observed an increased risk of postpartum depression in women who used epidural analgesia, although this topic remains controversial (39).

Domestic violence has been widely recognized as a significant risk factor for postpartum depression (40–42). Women who experience this type of violence are more likely to develop depressive disorders, especially in the first four weeks after birth (43). Psychological violence is as damaging as physical violence, although it is less frequently detected clinically (44). In this sense, the results of our study align with existing literature, showing that higher scores on the WAST scale, which indicates risk of partner abuse, are associated with an increased risk of PPD.

In our study, a lack of social support was identified as a significant risk factor for the development of postpartum depression and suicidal thoughts. For our assessment, we used two validated instruments: the Family APGAR and the MOS-SSS scale, which allowed us to analyze different dimensions of social support in a structured manner. Although we did not find previous studies that specifically use these scales, there is literature that supports this association through the use of other tools such as the J-SSQ scale (45), which has also observed that women with less social support more frequently present symptoms of PPD (46). These findings reinforce the idea that perceived social support is a protective factor against PPD and suicidal ideation, and the fact that we used validated scales in this study contributes to objectifying this relationship, underscoring the importance of assessing this aspect in perinatal care.

## 5 Strengths and limitations

It is important to note that the literature on risk factors for postpartum depression and suicidal ideation beyond the immediate postpartum period is limited. To date, this is the first study to use a validated scale to assess disrespectful treatment during birth and its relationship with the risk of postpartum depression and suicidal ideation beyond 6 months postpartum, providing novel evidence in this field. It is recommended that future research expand follow-up on mental health issues beyond the immediate postpartum period to better understand the long-term course of these disorders.

This study has some limitations. The sample was restricted to the Spanish population, which could limit the generalizability of the findings to other cultural contexts. The use of self-report scales may be research participants to response bias. As this is an observational study, the presence of confounding bias cannot be completely ruled out. However, an attempt was made to minimize its impact through multivariate analysis, so *a priori* its influence on the results would have been minimal. The sample size is limited due to the small number of women with suicidal ideation, which could affect the study's ability to detect associated risk factors. Finally, despite controlling multiple variables, some unaccounted confounding factors may have influenced the results.

## 6 Conclusions

In this study, two risk factors associated with postpartum depression were identified: hospital readmission and elevated scores on the CARE-MQ scale. Two protective factors were also identified: breastfeeding upon discharge and high scores on the MOS-SSS scale. Regarding suicidal ideation, hospitalization of the newborn was identified as a risk factor, while elevated scores on the family APGAR test acted as a protective factor for its onset.

The findings of this study highlight the importance of extending follow-up of women beyond the immediate postpartum period, with special attention to their mental health. It is recommended that healthcare professionals incorporate validated scales for detecting abuse and disrespect during childbirth, which can serve as effective tools for identifying women at risk for postpartum depression and suicidal ideation. On the other hand, it is essential to encourage the systematic implementation of screening for these disorders during postnatal consultations. At the political level, it would be advisable to promote and establish specific policies aimed at eliminating abuse and mistreatment during childbirth, including the implementation of institutional protocols for the prevention and detection of obstetric violence and the development of accountability mechanisms within healthcare settings. These measures, together with policies that guarantee a safe and respectful birth experience, would contribute to reducing the risk of postpartum depression and suicidal ideation, thereby protecting the physical and emotional well-being of mothers.

## Data Availability

The original contributions presented in the study are included in the article/Supplementary Material, further inquiries can be directed to the corresponding author.
